# Associations of vitamin D with lipid metabolism, inflammation, and mortality vary by glycemic status and gender: a nationwide prospective study

**DOI:** 10.3389/fnut.2025.1597527

**Published:** 2025-10-08

**Authors:** Meizhi Cai, Xuan Jiang, Xinyi Xu, Sidi Zhao, Yue Sun, Yushuo Yang, Ying Gu, Yifan Huang

**Affiliations:** ^1^Clinical Nutrition Division, The Second Affiliated Hospital of Soochow University, Suzhou, China; ^2^School of Public Health, Suzhou Medical College of Soochow University, Suzhou, China

**Keywords:** vitamin D, dyslipidemia, systemic inflammation, oxidative stress, cardiometabolic risk, gender differences, NHANES, glycemic control

## Abstract

**Background:**

We aimed to investigate the associations of 25-hydroxyvitamin D (25OHD) with lipid, inflammatory, and injury biomarkers, and their potential mediating roles in vitamin D-related mortality.

**Methods:**

We analyzed data from 4,144 participants in the 2015–2018 National Health and Nutrition Examination Survey (NHANES). Glycemic status was classified using fasting plasma glucose and hemoglobin A1c levels. Key biomarkers were assessed, including visceral adiposity index (VAI) and systemic inflammation response index (SIRI). Multivariate linear regression and restricted cubic spline models examined associations.

**Results:**

In females, 25OHD was positively associated with HDL-cholesterol across all glycemic subgroups, and with triglycerides and total cholesterol in the normal glycemic subgroup (all *p* < 0.05). In males, inverse associations of 25OHD with triglycerides and VAI were most pronounced in the prediabetes group (*p* < 0.05). 25OHD was inversely associated with SIRI in normoglycemic individuals and prediabetic males. Mediation analyses revealed that SIRI partially mediated the inverse association between 25OHD and mortality only in prediabetic males, while lipid and injury biomarkers showed no significant mediation effects in any subgroup.

**Conclusion:**

Gender and glycemic status influence the associations between vitamin D and biomarkers, with inflammation potentially mediating the relationship between vitamin D and mortality in prediabetic males.

## 1 Introduction

As a classic fat-soluble secosteroid hormone, vitamin D plays a crucial role in calcium homeostasis and bone metabolism. Nevertheless, growing evidence over the past decades indicates that vitamin D’s influence extends far beyond skeletal health, encompassing a wide array of physiological processes, including glucose-lipid metabolism, inflammation response, and immune function (1, 2). Observational studies and meta-analyses have linked vitamin D deficiency to an increased risk of various non-communicable chronic diseases, including cardiovascular disease (3), type 2 diabetes (4), certain cancers (5), and even all-cause mortality (6). Consequently, maintaining optimal vitamin D levels has been proposed as a potential strategy for improving overall health and preventing chronic diseases (7).

Despite growing recognition of its multiple effects, the precise mechanisms by which vitamin D influences metabolic health remain incompletely understood. In particular, the extent to which vitamin D modulates distinct biological processes such as lipid metabolism, systemic inflammation, and cellular injury, as well as whether these factors mediate its effects on long-term health and mortality, remains an area of active investigation. A substantial body of evidence indicates that the effects of vitamin D on metabolic health are significantly modulated by both gender and glycemic status (8, 9). Therefore, a central, *a priori* hypothesis of our study was that these factors would act as critical effect modifiers, making a stratified analysis a necessary, hypothesis-driven component of our research design. A nuanced understanding of these specific interactions is particularly important, as the progression from normoglycemia to diabetes is tightly linked to disturbances in adiposity and systemic inflammation—two core components of overall metabolic health.

To better characterize these complex relationships, researchers are increasingly moving beyond single-marker analysis to gain a more holistic view of biological processes. This trend is evident across diverse fields, employing strategies that range from reference-free transcriptomic analyses in oncology (10) to the use of composite indices in metabolic research, such as the visceral adiposity index (VAI). The VAI, which integrates waist circumference (WC), body mass index (BMI), triglycerides (TG), and high-density lipoprotein cholesterol (HDL-C), provides a robust measure of metabolic dysfunction (11). Similarly, the systemic inflammation response index (SIRI) and systemic immune-inflammation index (SII), derived from complete blood counts, offer valuable insights into an individual’s systemic inflammatory burden (12). However, the extent to which vitamin D is associated with these indices—and whether these associations vary by gender and glycemic status—remains largely unexplored.

Therefore, in this study, we aimed to examine the associations between vitamin D status and lipid, inflammation, and cellular injury biomarkers, including VAI, SII, and SIRI. Additionally, we sought to determine whether these factors mediate the relationship between vitamin D and mortality risk, with a particular focus on gender- and glycemia-specific differences.

## 2 Materials and methods

### 2.1 Study design and participant selection

**Figure 1:**
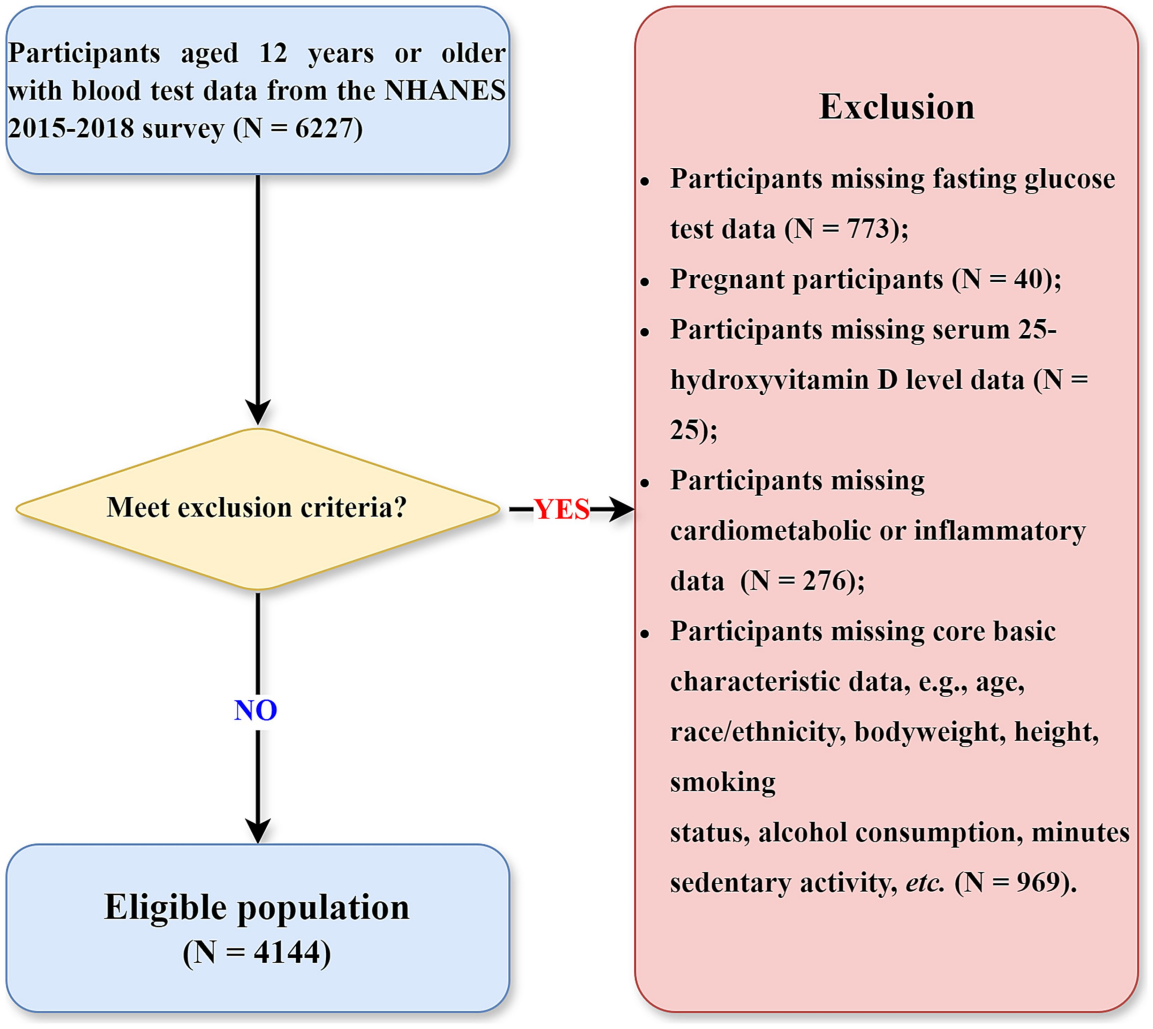
Flowchart of study population selection.

This study utilized baseline data from the National Health and Nutrition Examination Survey (NHANES) 2015–2018 cycles, with mortality outcomes ascertained prospectively through linkage to National Death Index (NDI) mortality files. This design allows for a temporal relationship to be established between baseline exposures and subsequent mortality. NHANES is a nationally representative program of studies designed to assess the health and nutritional status of adults and children in the United States, employing a complex, multistage probability sampling design (13). Data collection encompasses detailed questionnaires, standardized physical examinations, and extensive laboratory analyses. The flow chart of this study is presented in Figure 1. Initially, 6,227 participants aged over 12 years who provided blood samples were considered. Participants were excluded according to the following exclusion criteria: (1) absence of valid fasting glucose measurements (number = 773); (2) pregnancy status (number = 40); (3) missing serum 25-hydroxyvitamin D (25OHD) measurements (number = 25); (4) incomplete cardiometabolic or inflammatory biomarker data (number = 276); and (5) missing critical demographic or lifestyle information (age, race/ethnicity, anthropometric measurements, smoking status, alcohol consumption, or sedentary behavior; number = 969). Although the initial inclusion criterion allowed participants aged ≥ 12 years with available laboratory data, all individuals under 18 years of age were ultimately excluded due to missing data on key variables required for this analysis—such as fasting glucose, lipid and inflammatory markers, lifestyle factors, and mortality follow-up. As a result, the final analytic sample consisted of 4,144 adults aged 18 years and older.

### 2.2 Anthropometric and laboratory assessments

Body weight and height were measured by trained health technicians using standardized protocols. BMI was calculated as weight in kilograms divided by height in meters squared. WC was measured at the level just above the iliac crest using a flexible tape, with participants standing and breathing normally. Blood samples were collected following a standardized protocol and analyzed at laboratories certified by the Centers for Disease Control and Prevention. All assays were performed using validated enzymatic or hematological methods under rigorous quality control, as detailed in the NHANES Laboratory Procedures Manuals. Serum triglycerides and HDL-C were measured enzymatically using the Roche Modular P chemistry analyzer. The same platform was also used to measure injury markers including creatine phosphokinase (CPK), lactate dehydrogenase (LDH), alanine aminotransferase (ALT), aspartate aminotransferase (AST). Complete blood counts, including neutrophils, lymphocytes, monocytes, and platelets, were assessed using the Beckman Coulter DxH 800 hematology analyzer. All assays followed rigorous quality control and quality assurance procedures as outlined in the NHANES Laboratory Procedures Manuals. Full details of these examination and laboratory procedures are publicly available at https://www.cdc.gov/nchs/nhanes/ (Accessed on 29 August 2025).

### 2.3 Outcome assessment

Mortality status was determined by linkage of NHANES participants to the NDI through probabilistic matching using a combination of personal identifiers (e.g., Social Security number, name, date of birth). The publicly available Linked Mortality File (LMF) used in this study, which provides data through December 31, 2019, can be accessed at http://www.cdc.gov/nchs/linked-data/mortality-files/index.html (Accessed on 29 August 2025). From this file, we identified all-cause mortality as the primary outcome. The follow-up time was calculated from the date of the NHANES examination to the date of death or the end of the follow-up period (December 31, 2019), whichever came first.

### 2.4 Definition of diabetes status

Diabetes status was determined according to the American Diabetes Association criteria (14): diabetes was defined as a fasting plasma glucose (FPG) level ≥ 7.0 mmol/L or hemoglobin A1C (HbA1c) ≥ 6.5%; prediabetes was defined as 5.6 mmol/L ≤ FPG < 7.0 mmol/L or 5.7% ≤ HbA1c < 6.5%; and normoglycemic status was defined as FPG < 5.6 mmol/L or HbA1c < 5.7%.

### 2.5 Calculation of Indices

The following composite indices were calculated based on previously established formulas. SII, SIRI, and VAI are calculated as follows: SII = (platelet count × neutrophil count)/lymphocyte count (15); SIRI = (neutrophil count × monocyte count)/lymphocyte count (16); VAI (male) = (WC/[39.68 + 1.88 × BMI]) × (TG/1.03) × (1.31/HDL-C); VAI (female) = (WC/[36.58 + 1.89 × BMI]) × (TG/0.81) × (1.52/HDL-C) (17).

### 2.6 Statistical analysis

Baseline participant characteristics were summarized using descriptive statistics. Data are presented as numbers (percentages) for categorical variables or medians (interquartile ranges) for continuous variables. Categorical variables were compared using the chi-square (*χ*^2^) test, while continuous variables were compared using the Kruskal–Wallis H test.

#### 2.6.1 Multivariate linear regression

Associations between serum 25OHD levels and lipid, inflammatory, and cellular injury biomarkers were evaluated using multivariate linear regression models. The general form of the model is:


$$Y=\beta_{0}+\beta_{1}X_{25OHD}+\sum_{i=2}^{p}\beta_{i}X_{i}+\in$$

where *Y* denotes the dependent biomarker, *X*_25OHD_ is the serum 25OHD level, *X_i_* (*i* = 2, …, p) are adjustment covariates, *βᵢ* are the corresponding regression coefficients, *p* is the total number of predictors in the model, and *ϵ* is the random error term. Model 1 was adjusted for age, race/ethnicity, and BMI, and model 2 additionally adjusted for alcohol consumption, smoking status, and sedentary activity.

#### 2.6.2 Restricted cubic splines (RCS)

To assess the potential nonlinear association between serum 25OHD and mortality, we fitted RCS within a Cox proportional hazards model:


$$\log\left(HR\left(X\right)\right)=s\left(X\right)+\sum_{j=1}^{q}\gamma_{j}Z_{j}$$

where s(*X*) denotes the spline function of serum 25OHD, Z*_j_* is the *j*-th covariate, γ*_j_* its coefficient, and *q* is the number of covariates. This corresponds to the proportional hazards form *h*(*t*∣*X*, *Z*) = *h*_0_ (*t*) exp.{*s*(*X*) + ∑γ_j_ *Z_j_*}, where *h*_0_ (*t*) is the baseline hazard. We used three knots placed at the 10th, 50th, and 90th percentiles of the 25OHD distribution. Nonlinearity was tested using likelihood ratio tests, and the proportional hazards assumption was verified using Schoenfeld residuals.

#### 2.6.3 Mediation analyses

To investigate whether biomarkers mediated the effect of 25OHD (X) on mortality (Y), we applied a counterfactual-based mediation framework. The mediator (M) model was:


$$M=\alpha_{0}+\mathrm{aX}+\sum_{j=1}^{r}\delta_{j}W_{j}+\varepsilon_{M}$$

and the outcome model for time-to-event mortality was:


$$\log\left(T\right)=\beta_{0}+c^{\prime }X+bM+\sum_{j=1}^{r}\theta_{j}W_{j}+\sigma \varepsilon_{Y}$$

where *T* is the survival time, *Wⱼ* denotes the *j*-th covariate (indexed from 1 to *r*, the total number of covariates); *a* quantifies the effect of *X* on *M*; *b* reflects the effect of *M* on the log-survival time given *X*; and *c’* represents the direct effect of *X* on the log-survival time given *M*. The term *σ*ε_Y_ represents the error, where σ is the scale parameter of the survival distribution. Based on these models, which were fitted using a Weibull distribution in the *survreg* function, the average causal mediation effect (ACME) and average direct effect (ADE) were estimated using the *mediation* package in R with quasi-Bayesian simulations (1,000 iterations). The analysis allowed for an exposure-mediator interaction.

All statistical analyses were performed using R software (version 4.3.3). A two-sided *p*-value < 0.05 was considered statistically significant.

## 3 Results

### 3.1 Baseline characteristics of participants

As shown in Table 1, the study population comprised 1,391 normoglycemic, 2020 prediabetic, and 733 diabetic individuals. The median age increased significantly from 35.0 years in the normoglycemic group to 63.0 years in the diabetic group (*p* < 0.001). The proportion of females was significantly higher in the normoglycemic group (59.7%) compared with the diabetic group (46.2%; *p* < 0.001). Racial/ethnic composition also differed significantly, with a higher prevalence of non-Hispanic Whites among normoglycemic and prediabetic participants. Additionally, smoking prevalence was greater among diabetic participants (47.3%) compared with normoglycemic individuals (36.6%; *p* < 0.001), and BMI increased progressively across groups. Triglyceride was highest, while HDL-C was lowest in the diabetic group. Other laboratory indices—including SIRI, LDH, and ALT—were significantly elevated, whereas serum 25(OH)D levels were highest in the diabetic group.

**Table 1:** The baseline characteristics of NHANES 2015–2018 participants grouped by diabetes status.

Variable	Normal	Prediabetes	Diabetes	*p*-value
Number	1,391	2,020	733	
Age, years	35.0 (25.0–51.0)	54.0 (39.0–66.0)	63.0 (54.0–71.0)	<0.001
Gender				
Female	830 (59.7)	948 (47.0)	339 (46.2)	<0.001
Male	561 (40.3)	1,072 (53.1)	394 (52.8)	
Race/Ethnicity				
Mexican American	188 (13.5)	345 (17.1)	137 (18.7)	0.006
Other Hispanic	150 (10.8)	228 (11.3)	97 (13.2)	
Non-Hispanic White	516 (37.1)	705 (34.9)	224 (30.6)	
Non-Hispanic Black	291 (20.9)	418 (20.7)	164 (23.4)	
Other (including multiracial)	246 (17.7)	324 (16.0)	111 (15.1)	
Alcohol consumption				
Yes	1,096 (78.8)	1,599 (79.2)	560 (76.4)	0.286
No	295 (21.2)	421 (20.8)	173 (23.6)	
Smoking status				
Yes	509 (36.6)	885 (43.8)	347 (47.3)	<0.001
No	882 (63.4)	1,135 (56.2)	386 (52.7)	
Body mass index, kg/m^2^	26.0 (22.6–30.6)	28.9 (25.2–33.7)	31.2 (27.2–36.3)	<0.001
Sedentary activity, minute	300 (180–480)	300 (180–480)	300 (180–480)	0.176
Triglycerides, mmol/L	0.8 (0.6–1.2)	1.1 (0.7–1.6)	1.4 (1.0–1.9)	<0.001
Total Cholesterol, mmol/L	4.6 (4.0–5.3)	4.9 (4.2–5.6)	4.5 (3.9–5.3)	<0.001
HDL-C, mmol/L	1.4 (1.2–1.8)	1.3 (1.1–1.6)	1.2 (1.0–1.4)	<0.001
LDL-C, mmol/L	2.7 (2.2–3.3)	2.9 (2.4–3.5)	2.6 (1.9–3.2)	<0.001
VAI	0.9 (0.6–1.5)	1.3 (0.8–2.2)	1.9 (1.2–3.0)	<0.001
RDW, %	13.3 (12.9–13.9)	13.6 (13.1–14.3)	13.8 (13.2–14.6)	<0.001
hs-CRP, mg/L	1.34 (0.6–3.2)	2.1 (0.9–4.8)	2.9 (1.3–6.2)	<0.001
SIRI	0.87 (0.58–1.58)	1.00 (0.65–1.47)	1.19 (0.77–1.76)	<0.001
SII	406.30 (291.00–569.25)	427.83 (303.61–609.71)	459.00 (328.07–639.95)	<0.001
CPK, IU/L	107.0 (74.0–170.0)	115.0 (75.0–181.0)	101.0 (67.5–168.0)	<0.001
LDH, IU/L	133.0 (117.0–155.0)	142.0 (123.0–164.0)	140.0 (122.0–163.0)	<0.001
ALT, U/L	17.0 (13.0–24.0)	20.0 (15.0–28.0)	22.0 (16.0–31.0)	<0.001
AST, U/L	21.0 (17.0–25.0)	21.0 (18.0–26.0)	21.0 (17.0–27.0)	<0.001
25OHD, nmol/L	61.8 (44.8–78.9)	63.3 (46.3–83.1)	66.4 (45.8–87.3)	<0.001

Values are presented as count (percentage) or median (25–75 interquartile range). SII is calculated as the product of neutrophil count and platelet count divided by lymphocyte count. SIRI is calculated as the product of neutrophil count and monocyte count divided by lymphocyte count. 25OHD, 25-hydroxyvitamin D; ALT, alanine aminotransferase; AST, aspartate aminotransferase; CPK, creatine phosphokinase; HDL-C, high-density lipoprotein-cholesterol; hs-CRP, high-sensitivity C-reactive protein; LDH, lactate dehydrogenase; LDL-C, low-density lipoprotein-cholesterol; RDW, red cell distribution width; SII, systemic immune-inflammation index; SIRI, systemic inflammation response index; VAI, visceral adiposity index.

### 3.2 Associations of 25OHD with lipid, inflammation, and injury biomarkers

The associations between vitamin D and various lipid parameters were modified by both diabetes status and gender (Table 2). In normoglycemic females, 25OHD was positively associated with triglycerides, total cholesterol, and HDL-C (all *p* < 0.05). In prediabetes, 25OHD was positively associated with HDL-C in both genders, while inverse associations with triglycerides and VAI were evident only in males. Among diabetic participants, a consistent positive association between 25OHD and HDL-C was observed in females, with no significant associations in other lipid measures for either gender.

**Table 2:** The association of serum 25OHD levels with lipid biomarkers in NHANES 2015–2018 participants.

Diabetes status	Gender	Variable	Crude model		Adjusted model 1		Adjusted model 2	
			*β*	*P*	*β_1_*	*P_1_*	*β_2_*	*P_2_*
Normal	Female	Triglyceride, mmol/L	0.002	<0.001	0.001	0.046	0.001	0.030
		Total Cholesterol, mmol/L	0.007	<0.001	0.003	0.027	0.003	0.030
		HDL-C, mmol/L	0.004	<0.001	0.002	<0.001	0.002	<0.001
		LDL-C, mmol/L	0.002	0.015	0.000	0.955	0.000	0.907
		VAI	0.001	0.355	0.001	0.331	0.001	0.286
	Male	Triglyceride, mmol/L	0.000	0.687	0.000	0.864	0.000	0.950
		Total Cholesterol, mmol/L	0.001	0.650	−0.002	0.234	−0.002	0.206
		HDL-C, mmol/L	0.000	0.833	0.000	0.625	0.000	0.573
		LDL-C, mmol/L	0.001	0.633	−0.002	0.299	−0.002	0.263
		VAI	<0.001	0.844	0.001	0.436	0.002	0.372
Prediabetes	Female	Triglyceride, mmol/L	0.001	0.179	0.000	0.509	0.000	0.634
		Total Cholesterol, mmol/L	0.004	<0.001	0.000	0.936	0.000	0.882
		HDL-C, mmol/L	0.003	<0.001	0.001	0.004	0.001	0.006
		LDL-C, mmol/L	0.000	0.626	−0.001	0.339	−0.001	0.389
		VAI	−0.001	0.306	<0.001	0.785	<0.001	0.811
	Male	Triglyceride, mmol/L	−0.002	0.016	−0.003	0.003	−0.003	0.004
		Total Cholesterol, mmol/L	−0.002	0.138	−0.001	0.653	−0.001	0.640
		HDL-C, mmol/L	0.002	<0.001	0.002	<0.001	0.002	<0.001
		LDL-C, mmol/L	−0.003	0.002	−0.001	0.230	−0.001	0.232
		VAI	−0.005	<0.001	−0.004	0.003	−0.004	0.004
Diabetes	Female	Triglyceride, mmol/L	0.000	0.770	0.000	0.984	0.000	0.893
		Total Cholesterol, mmol/L	0.001	0.784	0.002	0.373	0.002	0.375
		HDL-C, mmol/L	0.003	<0.001	0.002	0.002	0.002	0.002
		LDL-C, mmol/L	−0.002	0.230	<0.001	0.874	0.000	0.858
		VAI	−0.004	0.097	−0.001	0.703	−0.001	0.798
	Male	Triglyceride, mmol/L	−0.001	0.447	−0.001	0.620	−0.001	0.604
		Total Cholesterol, mmol/L	−0.005	0.013	−0.001	0.620	−0.001	0.604
		HDL-C, mmol/L	0.001	0.171	0.000	0.681	0.000	0.664
		LDL-C, mmol/L	−0.005	0.003	−0.001	0.707	−0.001	0.687
		VAI	−0.003	0.392	0.001	0.845	0.001	0.872

The regression coefficients *β* and *p-*values are derived from linear regression analysis. Model 1, adjusted for age, race/ethnicity, and body mass index; model 2, adjusted for age, race/ethnicity, body mass index, alcohol consumption, smoking status, and minutes sedentary activity. 25OHD, 25-hydroxyvitamin D; HDL-C, high-density lipoprotein-cholesterol; LDL-C, low-density lipoprotein-cholesterol; VAI, visceral adiposity index. For models with VAI as the outcome, BMI was not included as an adjustment variable to avoid collinearity.

As presented in Table 3, in normoglycemic individuals, 25OHD was inversely associated with SIRI in females, and with both SII and SIRI in males after adjustment (all *p* < 0.05), while no significant association was found with high-sensitivity C-reactive protein (hs-CRP) or red cell distribution width (RDW) in certain subgroups. Similarly, in males, the serum 25OHD level was significantly and inversely associated with SII and SIRI after adjustment. In prediabetic males, the inverse association with SIRI remained significant (*p* = 0.032), whereas such associations were not observed in females. In the diabetic group, initial positive associations in males were attenuated after adjustment.

**Table 3:** The association of serum 25OHD levels with inflammation biomarkers in NHANES 2015–2018 participants.

Diabetes status	Gender	Variable	Crude model		Adjusted model 1		Adjusted model 2	
			*β*	*P*	*β_1_*	*P_1_*	*β_2_*	*P_2_*
Normal	Female	RDW, %	−0.005	0.002	−0.002	0.211	−0.002	0.199
		hs-CRP, mg/L	−0.013	0.043	−0.005	0.519	−0.004	0.546
		SIRI	0.000	0.499	−0.002	0.006	−0.002	0.008
		SII	−0.282	0.294	−0.335	0.281	−0.327	0.293
	Male	RDW, %	0.000	0.875	−0.001	0.386	−0.001	0.417
		hs-CRP, mg/L	−0.008	0.415	−0.012	0.278	−0.012	0.287
		SIRI	0.001	0.340	−0.004	0.006	−0.005	0.005
		SII	−0.298	0.464	−1.048	0.019	−1.142	0.011
Prediabetes	Female	RDW, %	−0.007	<0.001	−0.002	0.139	−0.002	0.137
		hs-CRP, mg/L	−0.035	<0.001	−0.016	0.112	−0.015	0.136
		SIRI	0.002	0.021	0.000	0.879	0.000	0.636
		SII	0.351	0.320	0.158	0.695	0.244	0.543
	Male	RDW, %	0.001	0.432	0.000	0.719	0.000	0.830
		hs-CRP, mg/L	0.001	0.876	−0.004	0.574	−0.003	0.747
		SIRI	0.002	0.105	−0.003	0.016	−0.003	0.032
		SII	0.637	0.050	−0.248	0.488	−0.206	0.564
Diabetes	Female	RDW, %	−0.003	0.182	−0.001	0.579	−0.002	0.479
		hs-CRP, mg/L	−0.042	0.075	−0.019	0.434	−0.022	0.361
		SIRI	−0.001	0.446	−0.003	0.086	−0.003	0.074
		SII	−0.528	0.357	−0.760	0.213	−0.786	0.199
	Male	RDW, %	−0.001	0.667	−0.003	0.233	−0.003	0.242
		hs-CRP, mg/L	−0.011	0.526	0.003	0.891	0.003	0.887
		SIRI	0.005	0.003	0.000	0.838	0.000	0.830
		SII	1.057	0.038	0.253	0.652	0.244	0.662

The regression coefficients *β* and *p*-values are derived from linear regression analysis. Model 1, adjusted for age, race/ethnicity, and body mass index; model 2, adjusted for age, race/ethnicity, body mass index, alcohol consumption, smoking status, and minutes sedentary activity. hs-CRP, high-sensitivity C-reactive protein; RDW, red cell distribution width; SII, systemic immune-inflammation index; SIRI, systemic inflammation response index.

The association between 25OHD and cellular injury is demonstrated in Table 4. Normoglycemic females exhibited a positive association between 25OHD and AST (*p* = 0.005). Normoglycemic males showed an inverse association between 25OHD and ALT in the adjusted model 1 (*p* = 0.042), however, this association lost significance upon further adjustment in the adjusted model 2 (*p* = 0.052). In prediabetic males and diabetic individuals, 25OHD was inversely associated with CPK in crude analyses, but these associations were not maintained after adjustment.

**Table 4:** The association of serum 25OHD levels with cellular injury biomarkers in NHANES 2015–2018 participants.

Diabetes status	Gender	Variable	Crude model		Adjusted model 1		Adjusted model 2	
			*β*	*P*	*β_1_*	*P_1_*	*β_2_*	*P_2_*
Normal	Female	CPK, IU/L	−0.129	0.396	0.188	0.286	0.177	0.317
		LDH, IU/L	0.099	0.008	0.036	0.383	0.024	0.560
		ALT, U/L	0.035	0.052	0.036	0.093	0.036	0.093
		AST, U/L	0.042	<0.001	0.033	0.007	0.034	0.005
	Male	CPK, IU/L	−0.305	0.414	0.867	0.033	0.788	0.054
		LDH, IU/L	0.019	0.737	0.014	0.819	−0.035	0.564
		ALT, U/L	−0.043	0.101	−0.058	0.042	−0.055	0.052
		AST, U/L	0.008	0.640	−0.010	0.611	−0.010	0.589
Prediabetes	Female	CPK, IU/L	−0.136	0.070	0.091	0.268	0.076	0.352
		LDH, IU/L	0.132	<0.001	0.081	0.056	0.056	0.070
		ALT, U/L	0.000	0.992	0.023	0.199	0.025	0.174
		AST, U/L	0.008	0.773	0.005	0.866	0.009	0.763
	Male	CPK, IU/L	−1.884	0.003	−0.628	0.381	−0.647	0.368
		LDH, IU/L	0.058	0.138	0.019	0.662	0.008	0.850
		ALT, U/L	−0.083	<0.001	−0.031	0.120	−0.030	0.132
		AST, U/L	−0.034	0.018	−0.021	0.189	−0.020	0.220
Diabetes	Female	CPK, IU/L	−0.321	0.029	−0.218	0.161	−0.222	0.154
		LDH, IU/L	0.005	0.933	−0.047	0.421	−0.057	0.322
		ALT, U/L	0.003	0.917	0.014	0.591	0.016	0.520
		AST, U/L	−0.003	0.903	−0.010	0.665	−0.009	0.709
	Male	CPK, IU/L	−0.682	0.018	0.112	0.711	0.107	0.725
		LDH, IU/L	0.075	0.207	0.084	0.204	0.086	0.192
		ALT, U/L	−0.051	0.106	0.023	0.501	0.022	0.526
		AST, U/L	−0.003	0.900	0.016	0.510	0.016	0.522

The regression coefficients *β* and *p-*values are derived from linear regression analysis. Model 1, adjusted for age, race/ethnicity, and body mass index; model 2, adjusted for age, race/ethnicity, body mass index, alcohol consumption, smoking status, and minutes sedentary activity. ALT, alanine aminotransferase; AST, aspartate aminotransferase; CPK, creatine phosphokinase; LDH, lactate dehydrogenase.

### 3.3 Vitamin D and total mortality

**Figure 2:**
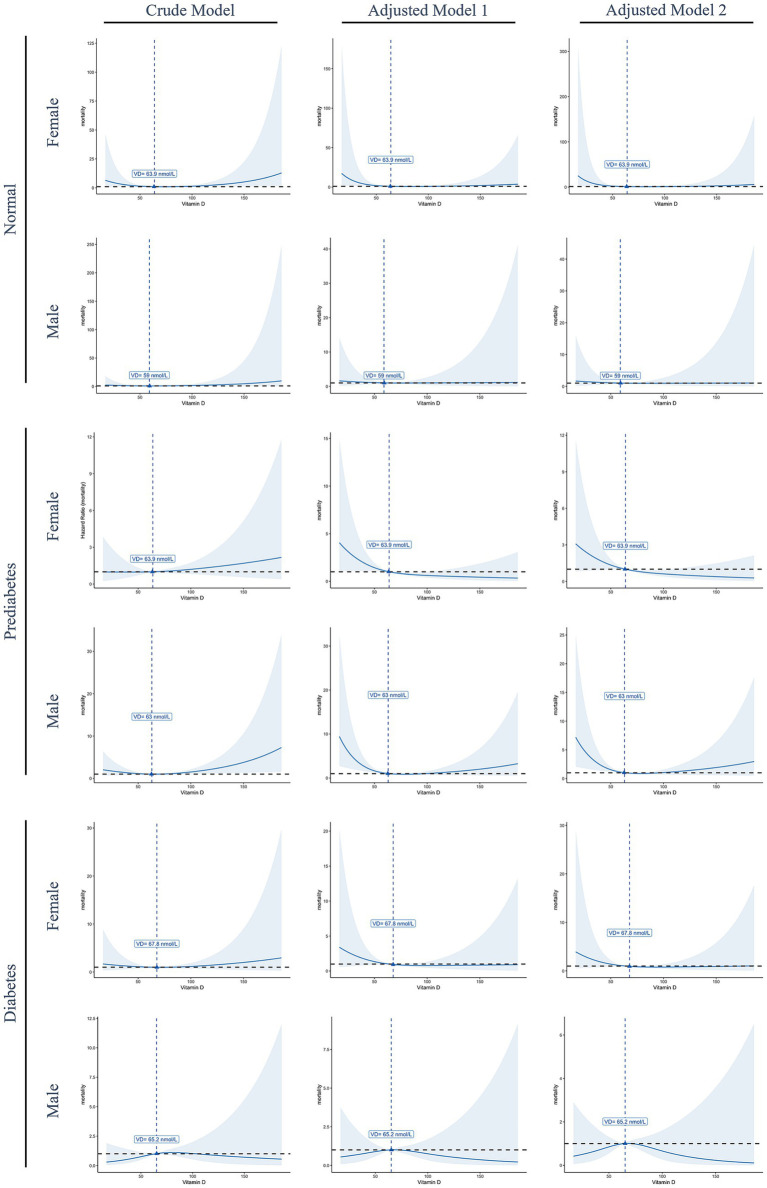
Restricted cubic spline curves illustrate the non-linear association between serum 25OHD levels and total mortality, stratified by gender and diabetes status. Solid blue lines represent the estimated hazard ratios (HRs), and shaded areas indicate 95% confidence intervals. The dashed horizontal black line denotes the reference value (HR = 1), while dashed vertical blue lines indicate the estimated threshold points where the slope of the association changes. Model 1, Adjusted for age, race/ethnicity, and body mass index; model 2, adjusted for age, race/ethnicity, body mass index, alcohol consumption, smoking status, and minutes sedentary activity.

Figure 2 presents the non-linear associations between serum 25OHD levels and total mortality across different diabetes statuses and genders. Among normoglycemic, prediabetic, and female diabetic participants, mortality risk declined with increasing serum 25OHD levels up to a certain threshold, after which the association plateaued. Notably, a statistically significant inverse association was observed only in male prediabetic participants. Although an inverted U-shaped relationship was observed in male diabetic participants, this association did not reach statistical significance.

### 3.4 Mediation analyses of lipids, inflammation, and injury

The results of mediation analyses are summarized in Tables 5–7. In normoglycemic and diabetic participants, lipid biomarkers did not significantly mediate the relationship between 25OHD and mortality in either gender. Among prediabetics, significant ADEs for total cholesterol, HDL-C, and low-density lipoprotein-cholesterol (LDL-C) were observed in females. For inflammatory markers, only prediabetic males exhibited a significant ACME for SIRI (*p* = 0.042), with no significant mediation observed in other groups. Similarly, mediation analyses for cellular injury markers revealed no significant mediation effects in normoglycemic or diabetic participants, with only prediabetic females showing significant ADEs in LDH, ALT, and AST.

**Table 5:** The mediation effect of lipid biomarkers in the association between 25OHD and mortality in NHANES 2015–2018 participants.

Diabetes status	Gender	Lipid biomarkers	ADE (95%CI)	*p*-value	ACME (95%CI)	*p*-value	Total effect (95%CI)	*p*-value
Normal	Female	Triglyceride, mmol/L	−1.18e-04 (−5.09e-04–0.00)	0.510	−4.84e-06 (−2.17e-05–0.00)	0.490	−1.23e-04 (−5.29e-04–0.00)	0.500
		Total Cholesterol, mmol/L	−1.18e-04 (−5.11e-04–0.00)	0.520	−4.65e-06 (−4.41e-05–0.00)	0.790	−1.23e-04 (−5.14e-04–0.00)	0.500
		HDL-C, mmol/L	−1.06e-04 (−5.13e-04–0.00)	0.640	−1.66e-05 (−7.12e-05–0.00)	0.380	−1.23e-04 (−5.20e-04–0.00)	0.590
		LDL-C, mmol/L	−1.23e-04 (−5.11e-04–0.00)	0.490	−5.70e-08 (−1.42e-05–0.00)	0.990	−1.23e-04 (−5.15e-04–0.00)	0.490
		VAI	−1.71e-04 (−5.59e-04–0.00)	0.410	−1.87e-06 (−9.79e-06–0.00)	0.570	−1.73e-04 (−5.62e-04–0.00)	0.400
	Male	Triglyceride, mmol/L	−1.03e-04 (−8.73e-04–0.00)	0.700	−5.12e-07 (−3.09e-05–0.00)	0.930	−1.03e-04 (−8.83e-04 –0.00)	0.700
		Total Cholesterol, mmol/L	−1.15e-04 (−8.83e-04–0.00)	0.780	1.15e-05 (−1.73e-05–0.00)	0.450	−1.03e-04 (−8.88e-04–0.00)	0.800
		HDL-C, mmol/L	−1.02e-04 (−8.84e-04–0.00)	0.780	−9.38e-07 (−6.89e-05–0.00)	0.960	−1.03e-04 (−8.75e-04–0.00)	0.800
		LDL-C, mmol/L	−1.18e-04 (−9.67e-04–0.00)	0.690	1.50e-05 (−1.54e-05–0.00)	0.410	−1.03e-04 (−9.24e-04–0.00)	0.720
		VAI	−8.96e-05 (−8.56e-04–0.00)	0.740	−2.28e-06 (−3.17e-05–0.00)	0.850	−9.19e-05 (−8.56e-04–0.00)	0.750
Prediabetes	Female	Triglyceride, mmol/L	−4.60e-04 (−9.04e-04–0.00)	0.056	8.97e-06 (−2.65e-05–0.00)	0.602	−4.51e-04 (−9.10e-04–0.00)	0.060
		Total Cholesterol, mmol/L	−4.48e-04 (−9.33e-04–0.00)	0.040	−2.89e-06 (−4.49e-05–0.00)	0.880	−4.51e-04 (−9.25e-04–0.00)	0.044
		HDL-C, mmol/L	−4.68e-04 (−9.15e-04–0.00)	0.024	1.77e-05 (−1.73e-05–0.00)	0.370	−4.51e-04 (−8.99e-04–0.00)	0.034
		LDL-C, mmol/L	−4.67e-04 (−9.38e-04–0.00)	0.038	1.62e-05 (−2.04e-05–0.00)	0.418	−4.51e-04 (−9.19e-04–0.00)	0.040
		VAI	−3.06e-04 (−6.78e-04–0.00)	0.080	1.94e-05 (−4.28e-06–0.00)	0.120	−2.87e-04 (−6.49e-04–0.00)	0.100
	Male	Triglyceride, mmol/L	−3.31e-04 (−9.76e-04–0.00)	0.320	1.90e-05 (−3.30e-05–0.00)	0.290	−3.12e-04 (−9.54e-04–0.00)	0.330
		Total Cholesterol, mmol/L	−3.21e-04 (−9.90e-04–0.00)	0.340	9.29e-06 (−2.73e-05–0.00)	0.660	−3.12e-04 (−9.69e-04–0.00)	0.350
		HDL-C, mmol/L	−3.09e-04 (−9.59e-04–0.00)	0.370	−2.53e-06 (−9.26e-05–0.00)	0.920	−3.12e-04 (−9.48e-04–0.00)	0.350
		LDL-C, mmol/L	−3.36e-04 (−1.02e-03–0.00)	0.320	2.40e-05 (−1.41e-05–0.00)	0.220	−3.12e-04 (−1.01e-03–0.00)	0.350
		VAI	−2.90e-04 (−9.86e-04–0.00)	0.360	6.42e-06 (−4.48e-05–0.00)	0.720	−2.83e-04 (−9.64e-04–0.00)	0.370
Diabetes	Female	Triglyceride, mmol/L	−4.65e-04 (−1.13e-03–0.00)	0.230	−5.95e-06 (−1.13e-04–0.00)	0.920	−4.70e-04 (−1.19e-03–0.00)	0.240
		Total Cholesterol, mmol/L	−4.74e-04 (−1.24e-03–0.00)	0.220	3.17e-06 (−4.76e-05–0.00)	0.840	−4.70e-04 (−1.24e-03–0.00)	0.230
		HDL-C, mmol/L	−6.71e-04 (−1.45e-03–0.00)	0.080	2.00e-04 (−4.33e-05–0.00)	0.200	−4.70e-04 (−1.27e-03–0.00)	0.210
		LDL-C, mmol/L	−4.72e-04 (−1.23e-03–0.00)	0.250	1.42e-06 (−4.40e-05–0.00)	0.970	−4.70e-04 (−1.21e-03–0.00)	0.250
		VAI	−5.49e-04 (−1.40e-03–0.00)	0.160	2.94e-05 (−3.28e-05–0.00)	0.350	−5.19e-04 (−1.39e-03–0.00)	0.180
	Male	Triglyceride, mmol/L	−3.33e-04 (−1.13e-03–0.00)	0.470	4.55e-06 (−4.94e-05–0.00)	0.890	−3.29e-04 (−1.13e-03–0.00)	0.470
		Total Cholesterol, mmol/L	−3.28e-04 (−1.18e-03–0.00)	0.410	−9.97e-07 (−5.45e-05–0.00)	0.960	−3.29e-04 (−1.19e-03–0.00)	0.400
		HDL-C, mmol/L	−3.36e-04 (−1.12e-03–0.00)	0.420	6.83e-06 (−4.72e-05–0.00)	0.800	−3.29e-04 (−1.10e-03–0.00)	0.430
		LDL-C, mmol/L	−3.29e-04 (−1.15e-03–0.00)	0.440	−2.81e-07 (−5.58e-05–0.00)	1.000	−3.29e-04 (−1.17e-03–0.00)	0.440
		VAI	−4.09e-04 (−1.22e-03–0.00)	0.360	−1.10e-06 (−4.38e-05–0.00)	0.940	−4.10e-04 (−1.23e-03–0.00)	0.350

25OHD, 25-hydroxyvitamin D; ACME, average causal mediated effect; ADE, average direct effect; CI, confidence interval; HDL-C, high-density lipoprotein-cholesterol; LDL-C, low-density lipoprotein-cholesterol; VAI, visceral adiposity index.

**Table 6:** The mediation effect of inflammation biomarkers in the association between 25OHD and mortality in NHANES 2015–2018 participants.

Diabetes status	Gender	Inflammation biomarkers	ADE (95%CI)	*p*-value	ACME (95%CI)	*p*-value	Total Effect (95%CI)	*p*-value
Normal	Female	RDW, %	−1.24e-04 (−4.99e-04–0.00)	0.480	8.94e-07 (−2.46e-05–0.00)	0.920	−1.23e-04 (−4.99e-04–0.00)	0.490
		hs-CRP, mg/L	−1.26e-04 (−5.15e-04–0.00)	0.510	2.75e-06 (−5.92e-06–0.00)	0.520	−1.23e-04 (−5.15e-04–0.00)	0.530
		SIRI	−1.02e-04 (−4.80e-04–0.00)	0.640	−2.09e-05 (−7.20e-05–0.00)	0.250	−1.23e-04 (−5.01e-04–0.00)	0.580
		SII	−1.24e-04 (−5.03e-04–0.00)	0.520	1.48e-06 (−8.69e-06–0.00)	0.820	−1.23e-04 (−5.00e-04–0.00)	0.520
	Male	RDW, %	−9.76e-05 (−9.12e-04–0.00)	0.740	−1.27e-05 (−7.15e-05–0.00)	0.870	−1.03e-04(−9.27e-04–0.00)	0.730
		hs-CRP, mg/L	−9.05e-05 (−8.43e-04–0.00)	0.810	−7.21e-05 (−2.69e-04–0.00)	0.380	−1.03e-04(−8.65e-04–0.00)	0.790
		SIRI	−1.43e-04 (−8.93e-04–0.00)	0.630	4.02e-05 (4.02e-05–0.00)	0.280	−1.03e-04 (−8.56e-04–0.00)	0.690
		SII	−1.60e-04 (−1.00e-03–0.00)	0.610	5.70e-05 (−1.29e-05–0.00)	0.150	−1.03e-04 (−9.44e-04–0.00)	0.690
Prediabetes	Female	RDW, %	−4.32e-04 (−9.22e-04–0.00)	0.082	−1.85e-05 (−5.03e-05–0.00)	0.158	−4.51e-04 (−9.48e-04–0.00)	0.074
		hs-CRP, mg/L	−4.50e-04 (−8.92e-04–0.00)	0.036	−4.38e-07 (−2.03e-05–0.00)	0.920	−4.51e-04 (−9.02e-04–0.00)	0.038
		SIRI	−4.63e-04 (−9.07e-04–0.00)	0.052	1.26e-05 (−5.79e-05–0.00)	0.720	−4.51e-04 (−8.88e-04–0.00)	0.054
		SII	−4.64e-04 (−9.11e-04–0.00)	0.040	1.36e-05 (−7.92e-05–0.00)	0.802	−4.51e-04 (−9.06e-04–0.00)	0.042
	Male	RDW, %	−3.05e-04 (−9.64e-04–0.00)	0.370	−6.67e-06 (−1.20e-04–0.00)	0.780	−3.12e-04(−9.77e-04–0.00)	0.350
		hs-CRP, mg/L	−3.02e-04 (−9.60e-04–0.00)	0.380	−9.75e-06 (−8.63e-05–0.00)	0.710	−3.12e-04 (−9.93e-04–0.00)	0.380
		SIRI	−2.42e-04 (−8.53e-04–0.00)	0.462	−7.01e-05 (−2.21e-04–0.00)	0.042	−3.12e-04 (−9.63e-04–0.00)	0.366
		SII	−3.01e-04 (−9.57e-04–0.00)	0.340	−1.06e-05 (−7.36e-05–0.00)	0.580	−3.12e-04 (−9.72e-04–0.00)	0.320
Diabetes	Female	RDW, %	−4.28e-04 (−1.17e-03–0.00)	0.270	−4.23e-05 (−1.73e-04–0.00)	0.450	−4.70e-04 (−1.22e-03–0.00)	0.230
		hs-CRP, mg/L	−4.55e-04 (−1.23e-03–0.00)	0.240	−1.55e-05 (−6.48e-05–0.00)	0.460	−4.70e-04 (−1.23e-03–0.00)	0.220
		SIRI	−4.29e-04 (−1.18e-03–0.00)	0.280	−4.13e-05 (−1.28e-04–0.00)	0.290	−4.70e-04 (−1.20e-03–0.00)	0.220
		SII	−4.63e-04 (−1.19e-03–0.00)	0.240	−7.52e-06 (−6.30e-05–0.00)	0.830	−4.70e-04 (−1.19e-03–0.00)	0.210
	Male	RDW, %	−2.57e-04 (−1.12e-03–0.00)	0.500	−7.21e-05 (−2.69e-04–0.00)	0.340	−3.29e-04 (−1.21e-03–0.00)	0.380
		hs-CRP, mg/L	−3.44e-04 (−1.13e-03–0.00)	0.440	1.47e-05 (−2.05e-04–0.00)	0.940	−3.29e-04 (−1.11e-03–0.00)	0.420
		SIRI	−3.08e-04 (−1.10e-03–0.00)	0.430	−2.12e-05 (−2.66e-04–0.00)	0.790	−3.29e-04 (−1.17e-03–0.00)	0.400
		SII	−3.56e-04 (−1.17e-03–0.00)	0.430	−2.7e-05 (−1.17e-04–0.00)	0.700	−3.29e-04 (−1.17e-03–0.00)	0.450

25OHD, 25-hydroxyvitamin D; ACME, average causal mediated effect; ADE, average direct effect; CI, confidence interval; HDL-C, high-density lipoprotein-cholesterol; LDL-C, low-density lipoprotein-cholesterol; RDW, red cell distribution width; SII, systemic immune-inflammation index; SIRI, systemic inflammation response index.

**Table 7:** The mediation effect of injury biomarkers in the association between 25OHD and mortality in NHANES 2015–2018 participants.

Diabetes status	Gender	Variable	ADE of Vit D and 95% CI	*p*-value	ACME of variables	*p-*value	Total effect	*p-*value
Normal	Female	CPK, IU/L	−1.23e-04 (−4.86e-04–0.00)	0.510	4.21e-07 (−1.18e-05–0.00)	0.950	−1.23e-04 (−4.84e-04–0.00)	0.500
		LDH, IU/L	−1.23e-04 (−5.04e-04–0.00)	0.490	5.89e-07 (−6.44e-06–0.00)	0.900	−1.23e-04 (−5.01e-04–0.00)	0.490
		ALT, U/L	−1.19e-04 (−5.19e-04–0.00)	0.520	−3.52e-06 (−4.62e-05–0.00)	0.310	−1.23e-04 (−5.42e-04–0.00)	0.490
		AST, U/L	−1.24e-04 (−5.00e-04–0.00)	0.550	1.46e-06 (−3.44e-05–0.00)	0.980	−1.23e-04 (−4.85e-04–0.00)	0.540
	Male	CPK, IU/L	−9.47e-05 (−8.47e-04–0.00)	0.780	−8.55e-06 (−6.00e-05–0.00)	0.660	−1.03e-04 (−8.44e-04–0.00)	0.760
		LDH, IU/L	−9.82e-05 (−8.61e-04–0.00)	0.700	−5.00e-06 (−4.05e-05–0.00)	0.810	−1.03e-04 (−8.60e-04–0.00)	0.700
		ALT, U/L	−9.12e-05 (−9.04e-04–0.00)	0.790	−1.20e-05 (−1.04e-04–0.00)	0.700	−1.03e-04 (−9.10e-04–0.00)	0.760
		AST, U/L	−7.80e-05 (−8.62e-04–0.00)	0.860	−2.52e-05 (−1.75e-04–0.00)	0.590	−1.03e-04 (−8.78e-04–0.00)	0.810
Prediabetes	Female	CPK, IU/L	−4.58e-04 (−8.99e-04–0.00)	0.058	7.14e-06 (−9.05e-06–0.00)	0.516	−4.51e-04 (−8.92e-04–0.00)	0.064
		LDH, IU/L	−4.65e-04 (−9.32e-04–0.00)	0.042	1.44e-05 (−8.05e-06–0.00)	0.242	−4.51e-04 (−9.16e-04–0.00)	0.048
		ALT, U/L	−4.49e-04 (−8.96e-04–0.00)	0.046	−2.14e-06 (−1.75e-05–0.00)	0.728	−4.51e-04 (−9.03e-04–0.00)	0.048
		AST, U/L	−4.51e-04 (−9.14e-04–0.00)	0.036	1.07e-07 (−3.48e-06–0.00)	0.738	−4.51e-04 (−9.10e-04–0.00)	0.040
	Male	CPK, IU/L	−3.11e-04 (−9.79e-04–0.00)	0.370	−8.44e-07 (−5.56e-06–0.00)	0.690	−3.12e-04 (−9.79e-04–0.00)	0.370
		LDH, IU/L	−3.16e-04 (−9.88e-04–0.00)	0.330	3.73e-06 (−9.78e-05–0.00)	0.960	−3.12e-04 (−9.84e-04–0.00)	0.320
		ALT, U/L	−2.88e-04 (−9.62e-04–0.00)	0.410	−2.43e-05 (−8.09e-05–0.00)	0.230	−3.12e-04 (−9.92e-04–0.00)	0.380
		AST, U/L	−2.83e-04 (−9.38e-04–0.00)	0.390	−2.86e-05 (−1.09e-04–0.00)	0.300	−3.12e-04 (−9.93e-04–0.00)	0.370
Diabetes	Female	CPK, IU/L	−4.69e-04 (−1.21e-03–0.00)	0.190	−1.91e-06 (−4.97e-05–0.00)	0.920	−4.70e-04 (−1.22e-03–0.00)	0.190
		LDH, IU/L	−4.42e-04 (−1.16e-03–0.00)	0.240	−2.89e-05 (−1.51e-04–0.00)	0.490	−4.70e-04 (−1.20e-03–0.00)	0.220
		ALT, U/L	−4.66e-04 (−1.17e-03–0.00)	0.220	−4.45e-06 (−3.86e-05–0.00)	0.750	−4.70e-04 (−1.18e-03–0.00)	0.220
		AST, U/L	−4.67e-04 (−1.20e-03–0.00)	0.240	−3.43e-06 (−2.62e-05–0.00)	0.990	−4.70e-04 (−1.19e-03–0.00)	0.230
	Male	CPK, IU/L	−3.14e-04 (−1.14e-03–0.00)	0.520	−1.51e-05 (−1.52e-04–0.00)	0.840	−3.29e-04 (−1.15e-03–0.00)	0.500
		LDH, IU/L	−3.12e-04 (−1.14e-03–0.00)	0.430	−1.7e-05 (−1.04e-04–0.00)	0.650	−3.29e-04 (−1.12e-03–0.00)	0.390
		ALT, U/L	−3.20e-04 (−1.23e-03–0.00)	0.440	−9.29e-06 (−7.32e-05–0.00)	0.620	−3.29e-04 (−1.22e-03–0.00)	0.430
		AST, U/L	−3.20e-04 (−1.18e-03–0.00)	0.440	−9.30e-06 (−7.15e-05–0.00)	0.710	−3.29e-04 (−1.17e-03–0.00)	0.420

25OHD, 25-hydroxyvitamin D; ACME, average causal mediated effect; ADE, average direct effect; ALT, alanine aminotransferase; AST, aspartate aminotransferase; CI, confidence interval; CPK, creatine phosphokinase; HDL-C, high-density lipoprotein-cholesterol; LDH, lactate dehydrogenase; LDL-C, low-density lipoprotein-cholesterol.

## 4 Discussion

This nationwide cohort study provides a novel and comprehensive exploration of the complex associations between vitamin D status, lipid metabolism, inflammation, cellular injury, and mortality, with a key focus on identifying gender-specific variations across different stages of glucose dysregulation. This nuanced approach allows for the identification of subgroups that may benefit most from targeted interventions in contrast to a one-size-fits-all approach.

The pronounced gender-specific associations we observed between vitamin D, lipid parameters, and inflammatory biomarkers are consistent with previous research, although specific findings across studies vary (18–20). For instance, Li et al. (20) reported that 25OHD levels were inversely correlated with LDL-C and total cholesterol (TC) in men aged over 50, but positively correlated with LDL-C and total cholesterol in women before age 50. In contrast, our study found that serum 25OHD was not associated with LDL-C in the overall population and was positively associated with total cholesterol only in normoglycemic females. These gender differences may be attributed to several factors. Firstly, sex hormones such as estrogen and testosterone play critical roles in lipid metabolism and immune regulation, which may influence how vitamin D exerts its biological effects. An animal study has shown that estrogen enhances vitamin D signaling by modulating hepatic estrogen receptor expression and vitamin D metabolic enzymes (21). In ovariectomized mice, concurrent deficiency of estrogen and vitamin D led to exacerbated hepatic steatosis, increased expression of lipogenesis-related genes [e.g., sterol regulatory element-binding protein 1c (SREBP1c), fatty acid synthase (FASn)], and elevated inflammatory cytokines [e.g., interleukin-6 (IL-6), tumor necrosis factor-*α* (TNF-α)], suggesting a synergistic role of estrogen and vitamin D in regulating lipid homeostasis and inflammation. Secondly, genetic factors may contribute to the observed disparities, as genes involved in vitamin D metabolism, vitamin D receptor (VDR) expression, and downstream signaling pathways might exhibit sex-specific polymorphisms or expression patterns. For instance, studies in rats have shown that the hepatic expression of *Vdr* (the gene encoding VDR) and *Cyp2r1* (a key 25-hydroxylase involved in vitamin D activation) is significantly higher in females compared with males (22). Such genetically determined differences in VDR levels and the activity of vitamin D metabolizing enzymes can logically lead to distinct intracellular vitamin D signaling capacities and quantitatively different physiological responses to vitamin D between sexes. Thirdly, differences in body composition, particularly body fat distribution, may affect vitamin D bioavailability since vitamin D is fat-soluble and can be sequestered in adipose tissue. Research by Cominacini et al. (23) indicates that visceral adipose tissue (VAT) particularly can trap vitamin D, thereby diminishing its metabolic availability. Their study also observed that distinct body fat distribution patterns in males and females correlated differently with vitamin D levels, suggesting these sex-specific adiposity characteristics contribute to differential vitamin D storage and bioavailability between men and women.

The association of vitamin D status with mortality risk among populations has been evaluated for several years; however, conclusions remain uncertain due to contradictory findings (24). In our study, mortality risk slightly declined with increasing serum 25OHD levels up to a threshold among normoglycemic, prediabetic, and female diabetic participants—aligning with several studies reporting inverse associations between vitamin D levels and mortality to a certain degree (6, 25–28). However, some studies have not found a significant association between blood 25OHD levels and all-cause mortality (29, 30). These discrepancies extend to intervention studies on vitamin D supplementation, where some studies reported beneficial effects on mortality (31, 32), while others did not (33, 34). Variations in study design, population characteristics, vitamin D dosage and form, as well as follow-up duration, may all contribute to these inconsistent findings (24, 34).

Vitamin D appears to exert its protective effects primarily by modulating immune pathways, such as regulating pro-inflammatory cytokines like TNF-α, IL-6, and interleukin-1β (IL-1β) (35). This regulation is often mediated through the VDR, which can interfere with pro-inflammatory signaling cascades, such as the nuclear factor-κB (NF-κB) pathway, a central regulator of cytokine gene expression (36). The potential links between vitamin D and lipid parameters, inflammation, cellular damage, and mortality prompted us to investigate whether these factors mediate the association between vitamin D and mortality. To mitigate the limitations of single biomarkers, we incorporated composite indices including VAI, SII, and SIRI for a more comprehensive assessment. Regarding the significant mediation by SIRI in prediabetic males, this finding should be interpreted with caution. Although the ACME was statistically significant, the effect size was modest, suggesting that SIRI may play only a limited role in the observed association. Therefore, the result should be viewed as a potential direction for future research rather than conclusive evidence of a clinically meaningful pathway. Larger-scale epidemiological research and well-designed experimental studies are warranted to further elucidate the underlying mechanisms and validate these findings across diverse populations.

Contrary to expectations, our mediation analyses revealed that neither lipid-related nor injury-related biomarkers significantly mediated the association between vitamin D and mortality across subgroups. This absence of mediation is particularly noteworthy, suggesting that inflammation may represent a more dominant and statistically detectable pathway than lipid modulation in the vitamin D–mortality relationship. Nevertheless, the lack of significant findings does not rule out potential involvement of these pathways, as previous studies have reported inconsistent results. For example, Huang et al. (37) found no association between plasma 25OHD and lipid metabolism markers, and reported no significant causal relationship. In contrast, Lhilali et al. (38) observed that low 25OHD levels were associated with elevated lipid profiles and atherogenic indices. Similar inconsistencies were found in studies exploring injury-related biomarkers. Bello et al.’s (39) meta-analysis concluded that vitamin D supplementation had no significant effect on LDH or creatine kinase, whereas Reichrath et al. (40) reported that average serum 25OHD levels ≥ 10 ng/mL were associated with significantly lower LDH levels, with a 3.86 U/L decrease per 1 ng/mL increase in 25OHD. Taken together, these findings highlight the heterogeneous and context-dependent nature of vitamin D’s biological effects, underscoring the need for further mechanistic investigations in diverse populations and clinical settings.

This study benefits from a large, nationally representative sample, allowing for robust analysis and generalizability to the broader United States population. By further stratifying our analysis by gender and glycemic status, we were able to identify important group-specific associations and different mediation pathways that would likely be missed in an analysis of the whole population. Taken together, our findings highlight why a ‘one-size-fits-all’ approach to vitamin D may be insufficient. Instead, our results support the need for a more personalized approach, where vitamin D interventions could be tailored to an individual’s specific characteristics, such as their gender and glycemic state, to achieve better health outcomes. Nevertheless, several limitations in our study still need to be considered. First and foremost, while mortality outcomes were ascertained prospectively, baseline vitamin D levels and various biomarkers—including potential mediators—were measured cross-sectionally. Therefore, the temporal order required for causal inference is lacking. As such, the observed associations and estimated mediation effects should be interpreted with caution. Future longitudinal studies assessing changes in both vitamin D status and biomarker profiles over time are warranted to further elucidate and confirm these potentially mediated relationships; additionally, the reliance on single time-point 25OHD measurements—common in many observational studies including NHANES—may not capture long-term vitamin D status. This introduces potential exposure misclassification due to seasonal fluctuations, supplement use, and lifestyle variation, which may not only bias internal estimates but also complicate comparison across studies. These limitations should be considered when interpreting the observed associations; furthermore, despite adjusting for key confounders, the potential for residual confounding remains. Our models could not fully account for several unmeasured variables, such as detailed dietary habits, specific physical activity patterns, or the precise dosage and duration of vitamin D supplementation. These lifestyle factors could correlate with both vitamin D status and cardiometabolic health, thereby potentially confounding the observed associations. Therefore, while our findings are robust to the included covariates, we cannot entirely exclude the possibility that these unmeasured lifestyle factors may have contributed to the observed associations; and finally, as all participants were from the United States, caution is warranted when generalizing the findings to other populations.

In conclusion, our study demonstrates that the associations of vitamin D with lipid metabolism, inflammation, and cellular injury are strongly influenced by gender and glycemic status. Systemic inflammation partially mediates the relationship between vitamin D and mortality in prediabetic males. Although further research is needed to confirm these findings, our study suggests that vitamin D intervention strategies may need to be tailored based on an individual’s gender and glycemic status.

## Data Availability

Publicly available datasets were analyzed in this study. This data can be found here: https://wwwn.cdc.gov/nchs/nhanes/; www.cdc.gov/nchs/linked-data/mortality-files/index.html.

## Glossary

25OHD: 25-hydroxyvitamin D
ALT: alanine aminotransferase
AST: aspartate aminotransferase
BMI: body mass index
CPK: creatine phosphokinase
FASn: fatty acid synthase
FPG: fasting plasma glucose
HbA1c: hemoglobin A1c
HDL-C: high-density lipoprotein cholesterol
hs-CRP: high-sensitivity C-reactive protein
IL-1β: interleukin-1β
IL-6: interleukin-6
LDH: lactate dehydrogenase
LDL-C: low-density lipoprotein cholesterol
NF-κB: nuclear factor-κB
NDI: national death index
NHANES: National Health and Nutrition Examination Survey
RDW: red cell distribution width
SII: systemic immune-inflammation index
SIRI: systemic inflammation response index
SREBP1c: sterol regulatory element-binding protein 1c
TC: total cholesterol
TG: triglycerides
TNF-α: tumor necrosis factor-α
VAI: visceral adiposity index
WC: waist circumference
